# The Hitchhiker's guide to glycoproteomics

**DOI:** 10.1042/BST20200879

**Published:** 2021-07-20

**Authors:** Tiago Oliveira, Morten Thaysen-Andersen, Nicolle H. Packer, Daniel Kolarich

**Affiliations:** 1 Institute for Glycomics, Griffith University, Gold Coast Campus, Gold Coast, Queensland, Australia; 2 Department of Molecular Sciences, Macquarie University, Sydney, New South Wales, Australia; 3 ARC Centre of Excellence for Nanoscale BioPhotonics, Griffith University, QLD and Macquarie University, NSW, Australia

**Keywords:** glycobiology, glycomics, glycoproteomics, mass spectrometry, multi-omics, proteomics

## Abstract

Protein glycosylation is one of the most common post-translational modifications that are essential for cell function across all domains of life. Changes in glycosylation are considered a hallmark of many diseases, thus making glycoproteins important diagnostic and prognostic biomarker candidates and therapeutic targets. Glycoproteomics, the study of glycans and their carrier proteins in a system-wide context, is becoming a powerful tool in glycobiology that enables the functional analysis of protein glycosylation. This ‘Hitchhiker's guide to glycoproteomics’ is intended as a starting point for anyone who wants to explore the emerging world of glycoproteomics. The review moves from the techniques that have been developed for the characterisation of single glycoproteins to technologies that may be used for a successful complex glycoproteome characterisation. Examples of the variety of approaches, methodologies, and technologies currently used in the field are given. This review introduces the common strategies to capture glycoprotein-specific and system-wide glycoproteome data from tissues, body fluids, or cells, and a perspective on how integration into a multi-omics workflow enables a deep identification and characterisation of glycoproteins — a class of biomolecules essential in regulating cell function.

## Protein glycosylation — the cells’ Swiss Army knife

Protein post-translational modifications (PTMs) enable the cell to produce profound structural and functional diversity from a limited number of protein-encoding genes [1]. Glycosylation plays an essential role across all domains of life [2]. Glycoproteins, together with other glycoconjugates, form the glycocalyx surrounding every living cell [3]. In this highly complex microenvironment, cell-surface receptors, signalling and cell adhesion molecules mediate and regulate cellular communication processes [4]. Intracellularly, *O*-GlcNAc glycosylation acts within the cytosol in a dynamic interplay with phosphorylation and is biosynthetically independent from the membrane and soluble glycoproteins trafficked to the extracellular environment after their formation [5,6].

In Eukaryotes, glycosylation is crucial for cell functions such as protein folding, regulating signalling or protein activity [2,7,8]. Congenital disorders of glycosylation (CDGs) are often embryonically lethal or phenotypically severe for affected individuals, emphasizing the essential role of glycosylation to life [2,9]. There are also examples of glycosylation ‘defects’ that do not impact normal development (e.g. human ABO blood groups [10]), which, however, can influence the susceptibility to infectious diseases and create crucial population diversity [11]. Changes in cell glycosylation have been associated with systemic pathologies such as (but not limited to) inflammation [12,13], cancer [14–19] or Alzheimer's disease [20,21]. Disease-associated changes in protein glycosylation are now considered a hallmark in many diseases, making glycans and glycoproteins promising molecular features with enormous diagnostic and prognostic value and potential therapeutic targets for precision medicine [22].

This review aims to provide an ‘easy-to-digest’ introduction to the analytical approaches relevant for studying protein glycosylation. For a comprehensive introduction to the diverse biological functions of protein glycosylation, readers are referred to the freely available *Essentials of Glycobiology* textbook [23]. Understanding the molecular basis of how glycans are involved in health and disease requires technologies to precisely determine both the glycan structures (glycomics), and characterise their location and structure at discrete sites on glycoproteins (glycoproteomics) expressed by a cell or in an entire organ, body fluid, tissue or organism of interest. While the literature harbours many excellent technical reviews covering specific aspects of glycomics (such as [24–34]) and glycoproteomics (examples include [35–44]) technologies and methodologies, there is a gap in the literature surveying the methods and practical issues of modern glycoproteomics relevant to beginners in the field. This mini-review intends to provide a concise introduction to the current strategies available to generate glycoproteomics data and to provide some guidance for designing tailored glycoproteomics experiments.

## Strategies to identify glycoproteins and their glycosylation features from complex samples

**Figure 1.**
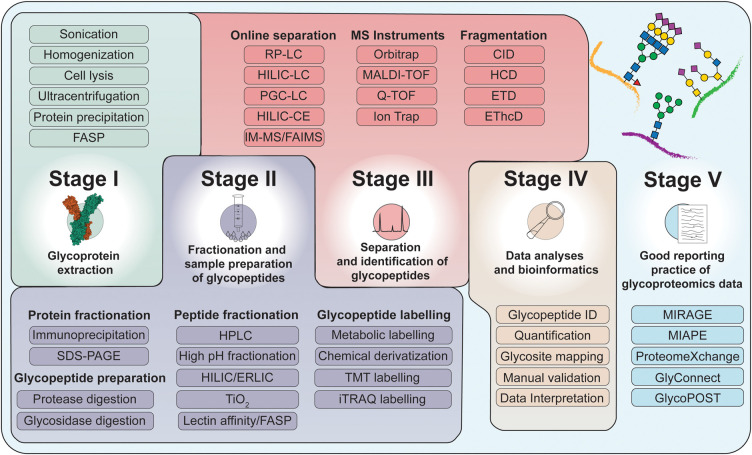
Schematic representation of the five key stages of a glycoproteomics experiment. *Stage I*: Extraction of glycoproteins from biological samples. *Stage II*: Proteolysis of glycoproteins, optional glycopeptide enrichment and labelling and offline fractionation to prepare the samples for MS analysis. *Stage III*: Online separation and fragmentation-based identification of glycopeptides. *Stage IV*: Bioinformatic (operator supervised) analyses of the data generated and integration of orthogonal data (e.g. glycomics data) to perform qualitative and quantitative glycoproteome profiling. *Stage V*: Data sharing and accurate reporting of experimental parameters provide a solid basis for integration with other *-omics* research and reuse in the glycoscience community.

There are five common stages in glycoproteomics analyses (Figure 1). Within each of these stages, a variety of techniques and tools are available that can be combined in different ways. The selection of specific tools will inevitably impact the generated data, as each technique comes with specific advantages and limitations that can impact the success of an experiment.

## Stage 1: glycoprotein extraction

The first step of any glycoproteomics experiment is to access the glycoproteins of interest from the complex biological matrix. Methods for tissue or cell lysis and protein extraction are diverse, and often depend on the type and available amount of the biological sample. Following tissue homogenisation or sonication and cell-disruption, the extraction step facilitates access to the (glyco)proteins of interest, in particular as many membrane glycoproteins require the presence of detergents to ensure sufficient solubility [45]. Established protocols using ultracentrifugation are available to enrich membrane glycoproteins [46] but sample amount is a limiting factor. In the case of frozen or formalin-fixed tissues, more intensive physical disruption techniques such as pressure-assisted extraction can provide better yields [38,47]. Protein extraction from body fluids is comparably straightforward, as these specimens already contain soluble glycoproteins. Please note that changing buffer and/or salt concentrations or depletion of highly abundant proteins, often used in proteomics experiments, can result in the unintended loss of glycoproteins at the sample preparation step [48].

**Figure 2.**
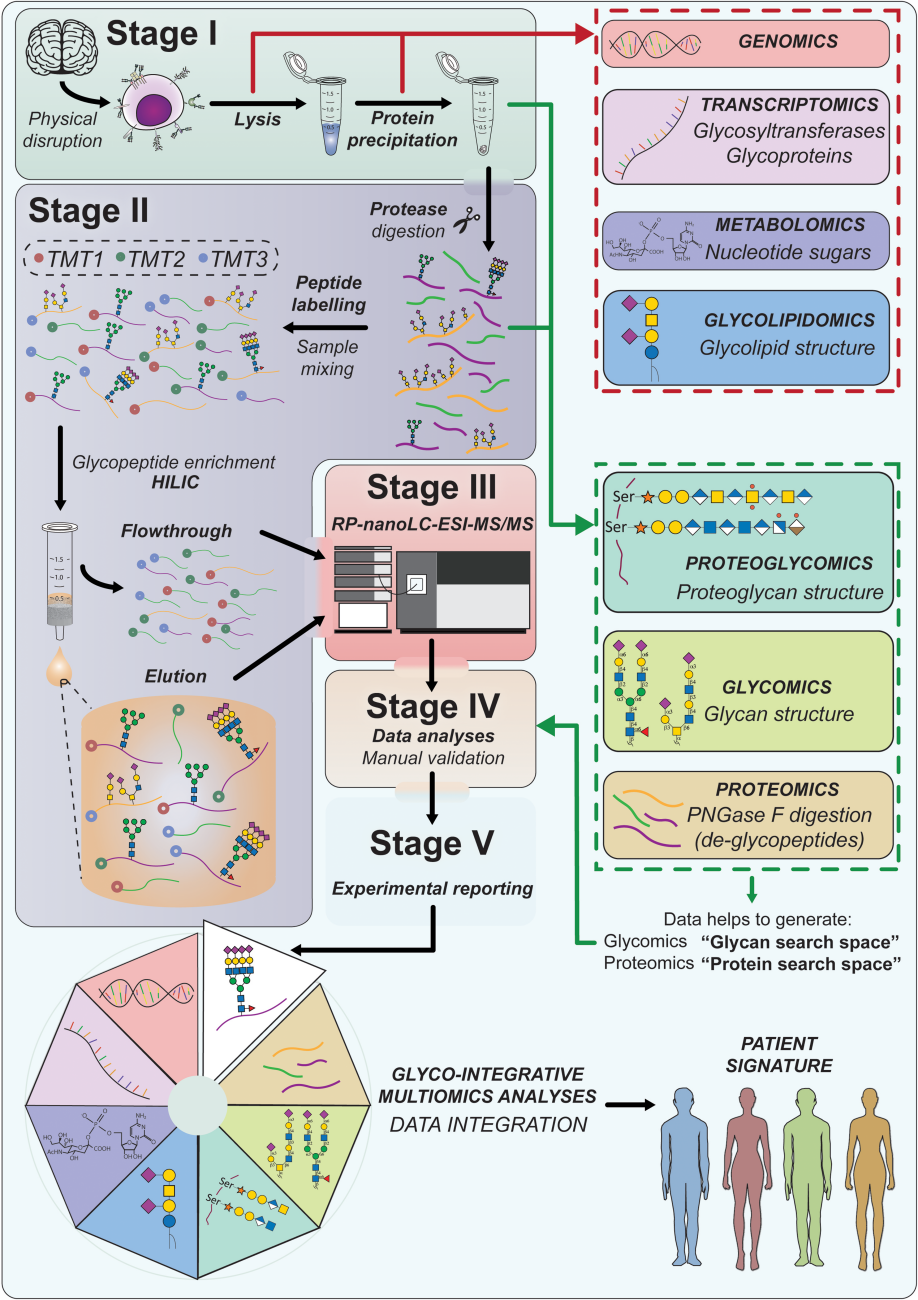
Example of an approach to integrate a representative glycoproteomics workflow into a multi-omics study. *Stage I*: After tissue lysis, material for genomic, transcriptomics or metabolomic analyses can be retrieved before the separation of lipids and glycolipids from glycoproteins for example by chloroform:methanol:water extraction. *Stage II*: Glycoproteins are digested using proteases, and can be either directly analysed (label-free proteomics) or subjected to labelling with, e.g. TMT-tags for quantitative glycoproteomics. Glycopeptide enrichment may be achieved by HILIC. *Stage III*: The enriched glycopeptides found in the eluate and the non-glycosylated peptides in the flowthrough fractions can be analysed by RP-nano-LC–ESI–MS/MS providing the data for *Stage IV*: Computational data analyses are performed using software tools such as Proteome Discoverer™ (Thermo Scientific) coupled to for example Byonic™ (Protein Metrics International, PMI) for protein and glycoprotein identification/quantitation. *Stage V*: Reporting and data sharing according to community guidelines and recommendations ensure lasting impact of outcomes. Integration of all data streams delivers a comprehensive picture of disease-associated effects for detection of diagnostic markers or therapeutic targets and for delivering novel fundamental understanding of cell function.

If other biomolecules such as DNA, RNA, metabolites, proteoglycans, glycosaminoglycans, lipids, glycolipids or glycans released from proteins are also to be analysed as part of a multi-omics study, the extraction conditions need to be adapted accordingly. In the case of glycolipids, for example, the frequently used chloroform–methanol precipitation method can be used to separate glycolipids from glycoproteins and other lipids [49,50] (Figure 2). Importantly, the composition and pH of the cell lysis buffer will also affect the solubility and integrity of the extracted glycoproteins, as will the presence of certain detergents, salts, denaturing agents and protease inhibitors. Finally, technologies such as filter-aided sample preparation (FASP), facilitate the use of complex MS-incompatible buffers for cell lysis, thus enabling the downstream processing of the extracted glycoproteins in Stage 2 (Figure 1) [51].

## Stage 2: fractionation and sample preparation of glycopeptides

In Stage 2, the extracted glycoproteins need to be prepared for downstream MS or LC–MS-based characterisation (see Stage 3). While there are some examples where top-down glycoproteomics of semi-purified, intact proteins is successfully applied to diagnose CDGs (reviewed in [52]) and native MS has been used to gain a more holistic view of multi-glycosylated proteins (e.g. myeloperoxidase [53] and neutrophil elastase [54]), bottom-up strategies using proteases are the most widely used approaches for clinical glycoproteomics. The two key steps in Stage 2 are (i) proteolytic digestion of the extracted glycoproteins and (ii) enrichment of glycopeptides/glycoproteins from the complex biological mixtures, though not necessarily in this order.

Intact glycoproteins or glycoprotein complexes can be fractionated by targeted affinity strategies such as immunoprecipitation, 2D gel electrophoresis, SDS–PAGE or lectin chromatography before proteolysis. Alternatively, glycopeptide enrichment is performed after the proteolytic digestion, or the glycopeptides may be pre-fractionated using conventional separation techniques to increase the analytical coverage of the sample of interest [42,55].

In system-wide glycoproteomics, glycopeptide enrichment remains an essential step since glycopeptides exhibit reduced ionisation efficiency in mass spectrometry compared with unglycosylated peptides, which is further aggravated by the intrinsic macro- and micro-heterogeneity of their glycan moieties [38,56]. Together, in un-enriched samples, these factors result in lower glycopeptide signal intensities relative to their non-glycosylated counterparts and a risk that these molecules are not being selected for fragmentation analyses.

### Proteolytic digestion of complex glycoprotein mixtures

Proteases are the ‘scissors’ used in bottom-up glycoproteomics experiments, producing a mixture of glycopeptides and peptides [57,58]. Trypsin is the most widely used protease due to its high specificity, availability, and efficiency over a range of conditions (e.g. pH, salts, detergents) [59,60]. As an additional benefit, the resulting C-terminal arginine/lysine residues carry a positive charge, enhancing the ionization and fragmentation of (glyco)peptides [61]. Other proteases such as chymotrypsin, endoproteinase Glu-C, Asp-N and Lys-C, are equally useful for comprehensive glycoproteomics, due to their complementary cleavage specificities [62]. Using dual-protease approaches often increases glycoprotein identification and sequence coverage for the in-depth characterisation of the glycoproteome [63–65].

However, these proteases often inefficiently digest mucin or mucin-like glycoproteins [41,66,67]. Their dense glycosylation makes their already few conventional protease cleavage sites in mucin-domains less accessible, posing unique challenges for successful MS analysis [68]. Excitingly, a suite of novel proteases, the so-called mucinases, have recently become available that facilitate the glycoproteomics analysis of mucins [44]. For example, the OpeRATOR® *O-*protease requires the presence of an *O*-glycan on a serine/threonine residue to cleave N-terminally before this site of glycosylation. Thus, this cleavage preference generates peptides that feature N-terminal *O*-glycosylation, and its application has resulted in the successful mapping of approximately 3000 *O*-glycosites [69,70]. Nevertheless, OpeRATOR® does not always follow this cleavage pattern and it is strongly advised to perform additional glycopeptide sequencing to confirm glycosylation site localisation. The secreted protease of C1 esterase inhibitor (StcE), a zinc metalloprotease [71], has also recently been used to generate *O*-glycopeptides from mucins, which significantly improved *O*-glycosite mapping [68].

Generally, glycoproteomics workflows rely on proteases with defined specificity such as the ones mentioned above. However, multiprotease mixtures such as Pronase, or proteases with very broad substrate specificity such as Proteinase K, can be of value when characterising single glycoproteins or simple mixtures [72,73]. These proteases exhibit a broad substrate specificity to produce glycopeptides with variable lengths (down to a single amino acid residue), which can be useful for the in-depth analysis of purified glycoproteins and to cover otherwise difficult-to-access regions within a protein. Such broadly specific proteases generate extremely heterogeneous glycopeptide mixtures, making them unsuitable for the analysis of complex samples.

### Enrichment using glyco-epitope binding agents

Enrichment strategies that take advantage of the presence of the glycan moiety are fundamental to improve glycoproteome coverage [42,74]. Antibodies, lectins or comparable binding agents (e.g. aptamers) have found widespread application to enrich or fractionate complex mixtures of glycoproteins or glycopeptides [45,75–77]. Plant-derived lectins are the most widely used agents for this purpose, but these often show reactivity to several different glyco-epitopes, particularly in the presence of a large dynamic range of glyco-structures [78]. As a consequence of the broad binding affinity patterns of most lectins, any conclusions about the nature of the enriched glyco-structures need to be carefully considered and ideally backed with additional experiments (e.g. glycomics) that provide a higher level of compositional and structural information [79].

Lectin affinity chromatography (LAC) has been used to successfully enrich protease-produced glycopeptides with short *O*-GalNAc structures such the ones derived from glycoengineered *SimpleCell* lines [80,81] or on cytosolic *O*-GlcNAc glycoproteins [6,82]. Chemical strategies based on releasing the glycans with simultaneous labelling of *O*-GlcNAc glycosylation sites, followed by thiol-Sepharose affinity-enrichment of these modified peptides have also been successfully employed for *O*-GlcNAc glycoproteomics [83]. Multi-LAC, the combination of two or more lectins within one column, has also been successfully employed to enrich glycopeptides for glycoproteomics experiments [42].

Probes based on bacterial and human lectins or specific anti-glycan antibodies generally appear to exhibit affinity to more specific glyco-epitopes than plant-derived lectins, but their commercial availability can be limited [84]. Unfortunately, the quality and purity of these agents varies drastically between vendors, and many show considerable levels of impurities that can jeopardise the interpretation of glycoproteomics experiments (Kolarich D, personal observations).

### Physicochemical agents for the enrichment of glycopeptides

A variety of enrichment strategies are based on non-biological reagents that target the physicochemical properties of the glyco-moieties of glycopeptides such as hydrophilicity, size, negative charge or the chemical properties of specific monosaccharides. These include approaches such as acetone precipitation [85,86], titanium dioxide (TiO_2_) for the enrichment of sialylated glycopeptides [87–89], boronic acid functionalised beads [90,91], electrostatic repulsion-hydrophilic interaction chromatography (ERLIC) [92,93] or the widely used hydrophilic interaction liquid chromatography (HILIC) [55,94–96]. These technologies enable the enrichment of intact glycopeptides, and in some cases different subsets of intact glycopeptides. The hydrazine coupling approach widely used for *N*-glycopeptide enrichment [97–99] is not suitable for intact glycopeptide analysis, as the glycan components remain covalently attached to the hydrazine beads. Peptides are enzymatically released using peptide *N-*glycosidase F (PNGase F), inevitably leading to a loss of structural information of the formerly attached glycan moiety. For more details on the many methods available for glycopeptide enrichment, the readers are referred to several excellent reviews on this topic [35,42,100–104].

No enrichment strategy (or combinations thereof) can quantitatively capture all glycopeptides in complex mixtures, and enrichment also results in the loss of quantitative information on site occupancy levels. Thus, compromises between selectivity and enrichment efficiency will have to be made based on the specific experimental aims of a project.

### How glycosidases can support a glycoproteomics experiment

*N*-glycans can be enzymatically removed using the hydrolytic enzyme PNGase F that efficiently cleaves between the innermost GlcNAc residue of all types of *N*-glycans [105], unless these contain an α1–3 linked core fucose residue as frequently found in plants and invertebrates [106], or are present in truncated forms (e.g. GlcNAc, GlcNAc-Fuc) [107,108]. PNGase F has frequently been used after enrichment as a strategy to reduce sample complexity and facilitate downstream proteomics analyses [109]. The enzymatic release of *N*-glycans by PNGase F converts asparagine to aspartate, and care should be taken to avoid misinterpretation of this conversion with the same mass increment (+0.98402 Da) induced by spontaneous deamidation that frequently occurs on asparagine residues in an asparagine–glycine (…NG…) sequon [76,110,111]. The use of heavy water during the PNGase F de-glycosylation reaction can introduce an ^18^O into the newly generated aspartate residue, and be used to discriminate spontaneous deamidation from de-glycosylation of asparagine residues [112,113].

Applying de-glycosylation enzymes with different specificities can avoid such false positive identifications of glycosylation sites while indirectly providing some limited, but still useful structural information. Combining endo-β-*N*-acetylglucosaminidase (Endo) H (which only cleaves oligomannosidic type *N*-glycans between the two GlcNAc residues of the chitobiose core, leaving a single GlcNAc attached to the glycopeptide) and PNGase F, using ^18^O-labeling, Cao and co-workers screened and successfully quantified site occupancy levels on HIV gp120 [64]. This approach enables relative quantitation of the macroheterogeneity since the resulting peptides, de-glycosylated peptides and single GlcNAc carrying glycopeptides exhibit similar ionisation efficiencies [56].

In contrast with these endoglycosidases, exoglycosidases digest specific terminal monosaccharide residues from glycans, providing an opportunity to gain insights into key structural features including biologically relevant glyco-epitopes. Exoglycosidase-assisted glycopeptide analysis can be used to determine the level of e.g. α2–3 linked NeuAc residues in a protein- and site-specific manner [114], or unambiguously determine the presence of sialyl Lewis X epitopes on specific glycans attached to specific sites when performed on glycopeptides from isolated glycoproteins [115,116]. However, such strategies are still to be applied at the glycoproteome-wide scale.

### Glycopeptide labelling strategies can facilitate quantitation and glycopeptide enrichment

Peptide labelling approaches such as tandem mass tags (TMT) [117–119] or isobaric tags for relative and absolute quantification (iTRAQ) [120,121] have been successfully employed in quantitative glycoproteomics workflows [122–126]. TMT provides accurate relative MS2 (or MS3) -based quantitation and an opportunity for multiplexing to reduce instrument time. Furthermore, it also improves the ET(hc)D fragmentation of glycopeptides by increasing the charge density of labelled glycopeptides [127]. TMT labelling strategies are easily implementable into any clinical glycoproteomics workflow but add sample handling steps and increase costs.

Metabolic derivatization methods are available based on the incorporation of isotopically labelled amino acids (e.g. SILAC) [128–130] (polypeptide-centric labelling) or the incorporation of non-natural monosaccharides [131,132] (glycan-centric labelling) into glycoproteins produced by cultured cells. Monosaccharide-specific click chemistry [133] has been leading the field, where monosaccharides modified with otherwise inert azido-groups replace the natural monosaccharides occurring within the cell. These are eventually incorporated into glycoconjugates and facilitate their enrichment and *in situ* visualisation. If used for enrichment, the glyco-moiety is frequently removed from the peptide for subsequent de-glycosylated peptide analysis [134]. A limitation of these strategies is the variable incorporation efficiency, often generating unlabelled glycoconjugates, as well as the impact of the labelling conditions on cell growth and physiology. Importantly, these reagents have been optimised with respect to applicability *in vivo* by significantly reducing their cellular toxicity [135]. Even though click chemistry metabolic labelling is a helpful tool for *in vitro* and animal model focussed glycoproteomic studies [136], its implementation into clinical glycoproteomics in the foreseeable future is unlikely.

A variety of different labelling strategies have been developed for the characterisation of glycoprotein mixtures of low complexity. Generally, these aim to either increase ionisation efficiency and/or to stabilise specific glycosylation features. Permethylation, a chemical derivatisation method, has been applied to glycopeptides from isolated glycoproteins to obtain more detailed glycan structural information [137]. However, this modification significantly increases the overall hydrophobicity of glycopeptides, which complicates their separation by reversed phase (RP) chromatography, making them unsuited for glycoproteome-wide analysis. Other forms of glycan derivatisation such as methylamidation have been successfully employed to derivatise and stabilise labile sialic acid residues for MALDI-TOF based glycopeptide profiling [138]. A sialic acid labelling approach using different stabilisation reagents can also be used to distinguish α2–3 from α2–6 linked sialic acid linkages at the glycopeptide level [139].

While these derivatisation strategies have unique benefits, chemical modifications can lead to unexpected reactions that unintentionally increase overall sample heterogeneity, subsequently affecting data analyses. Hence, it is important to consider the benefits and limitations before including glycopeptide labelling in the experimental design.

## Stage 3: separation and identification of glycopeptides

Most glycoproteomics workflows use advanced online nano-scale separations such as nano-flow liquid chromatography (nano-LC) or electrokinetic separation (e.g. capillary electrophoresis, CE) coupled with ESI–MS to detect and characterise intact glycopeptides. The past decade has seen tremendous advancements in both off- and online separation and detection technologies that have increased the sensitivity, accuracy and throughput of glycoproteomics workflows [140,141]. It would go beyond this mini-review to provide a detailed account on the many different aspects of MS-based glycopeptide separation and detection, hence here we are focussing on a high-level overview of the most important advantages and challenges of widely used techniques. For more details, we refer readers to recent literature on this topic [38–40,44,142].

### MS-coupled separation techniques for glycoproteomics

In principle, any ultra- or high-performance liquid chromatography (UPLC/HPLC) based separation method that can be performed using MS-compatible solvents can be used to separate complex glycopeptide mixtures prior to MS analysis. RP-LC is without doubt the most widely used separation technique for this purpose due to its unmatched peak capacity, versatility, simplicity and robustness [64,128,143]. A wide selection of different RP materials is available, with a variety of additional separation functionalities, such as improved retention of more hydrophilic compounds (such as glycopeptides) or better separation capacity resulting in reduced LC peak width and thus improved MS signal intensities. In principle, the same LC conditions used for peptide separation can also be employed for the separation of glycopeptides.

Glycopeptides are usually less hydrophobic than their non-glycosylated counterparts, and very hydrophilic glycopeptides might not exhibit sufficient interaction with the RP-matrix when loading the sample in low concentrations of organic solvent as it is commonly done in standard peptide RP-LC. Using pre- and analytical columns optimised to work under completely aqueous conditions can help to capture such very hydrophilic glycopeptides [57], particularly in the case of mucin-type glycopeptides where many sites of glycosylation can be occupied within a single glycopeptide [144]. The loss of hydrophilic glycopeptides can also be minimised by combining different stationary phases such as C18-RP and porous graphitized carbon (PGC), where the latter captures hydrophilic glycopeptides from the RP flowthrough before glycopeptides from both columns are consecutively eluted for MS analyses [73].

Another attractive separation technique, though less widely used, is based on CE separation that can provide excellent separation and sharp peaks with high signal-to-noise ratios [145,146]. CE offers the capacity to separate distinct glycoforms attached to peptides (e.g. linkage difference of sialic acids) based on their charge and physical characteristics [147]. However, the online coupling of CE separation to MS instruments can be challenging due the limited number of MS-compatible electrolytes necessary for electromigration [148], and CE is less frequently employed than LC. Recent advances have made CE-nanoESI systems commercially available. While this separation strategy remains to be applied for system-wide glycoproteomics, its capacity to separate glycans and glycopeptides of purified proteins has recently been shown [147,149,150].

Nano-LC can alternatively be coupled with MALDI-TOF-MS detection, which has the advantage of having the MS analyses decoupled from the LC step, facilitating the re-analyses of each spot at a later time if necessary. This has found some applications for glycoprotein-centric analyses such as IgM [151], or chemically glycosylated vaccine candidate glycoproteins such as cross-reactive material 197 (CRM^197^) [152]. While several publications describe this system for shotgun clinical proteomics (e.g. [153]), we are not aware of studies that have used LC-MALDI-TOF-MS for glycoproteomics.

Ion-mobility MS (IM-MS) is a recent technology that shows promise for improved analysis of glycopeptides [154,155]. IM-MS is a gas-phase separation method of ions based primarily on their mass and charge but also their size and shape [156]. Given that glycopeptides are usually considerably larger and thus occur in higher charge states than unglycosylated co-eluting peptides, IM-MS provides an opportunity to separate glycopeptides from unglycosylated peptides within an online experiment using field asymmetric ion mobility spectrometry (FAIMS) [157]. While these recent technology developments have not yet found widespread application in glycoproteomics [44], promising data have been published for the characterisation of isolated glycoproteins [158–160], where IM-MS has been reported to be able to distinguish sialic acid linkage isomers (α2–3 or α2–6) from otherwise isobaric glycopeptide precursors [161], and to enable characterisation of isomeric glycopeptides where different sites on the same peptide are glycosylated [162].

### Glycopeptide fragmentation — destructive approaches to decipher glycoproteomes

Identification of glycopeptides from complex samples would be impossible without fragmentation techniques optimised to deliver information on both the peptide and glycan moieties [163–165]. In combination with MS analysers that acquire product ion spectra with high mass accuracy across a wide *m/z* range, the fragmentation of glycopeptides may in favourable cases generate sufficient product ion information to facilitate software-assisted identification [166,167]. Glycopeptide fragmentation is perhaps one of the most central aspects within a glycoproteomics experiment, as it generates the fragment ‘fingerprint’ of a specific glycopeptide that is then used to determine the composition of the glycan and the sequence of the peptide. Depending on the type of fragmentation, the site of glycosylation can also be determined from the same product ion spectrum [39,168,169] (Table 1). Hybrid-type MS instruments (e.g. Orbitrap Tribrid) can perform different fragmentation schemes in parallel [164] and excellent reviews have thoroughly discussed the pros and cons of current fragmentation technologies [39,163,165]. Hence, the selection of the fragmentation scheme most suitable for each experiment is crucial to generate informative product ions of both the peptide and glycan moieties. Here, we limit the discussion to the fragmentation technologies most commonly used in glycoproteomics (Table 1).

**Table 1.** Overview on the most common fragmentation techniques in glycoproteomics

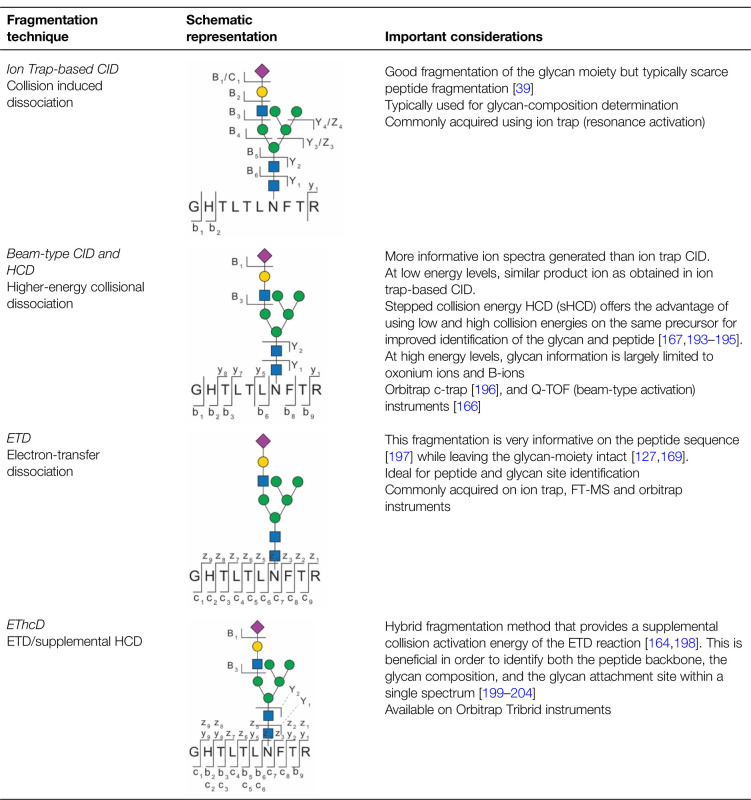

The choice of fragmentation scheme depends on (i) instrument availability, (ii) specific aims of an experiment and (iii) available sample amount. Each technique has specific advantages and limitations that need to be balanced based on the individual project aims.

Collision-induced dissociation (CID) techniques are often used in glycoproteomics but fundamentally differ if performed on ion trap (resonance-type activation) or Q-ToF (beam-type activation) instruments. In principle, both CID-types result largely in fragmentation of the glycan backbone but leave the peptide backbone relatively intact when performed at lower excitation levels sufficient to fragment non-modified peptides (Table 1).

Increasing the energy to achieve higher-energy collisional dissociation (HCD) may result in the generation of sufficient peptide produced ions that facilitate peptide sequence assignment, next to glycan oxonium product ions. Stepped-HCD (sHCD), where the fragmentation energies are being modulated from low to high, delivers more balanced product ion spectra that usually contain more information on both the glycan and peptide moiety of glycopeptides [166].

These fragmentation techniques rarely deliver reliable information on the site of glycan attachment, as achieved by ion-induced dissociation techniques (e.g. electron-transfer dissociation (ETD), electron-capture dissociation (ECD)) [41]. While in most cases these are not necessary for site assignment of *N*-glycopeptides due to the well-known *N*-glycosylation sequon (**N**-X-S/T/C; X≠P) [111], these fragmentation methods become really important when the modification site cannot readily be predicted, such as in *O*-glycosylation or chemical glycosylation reactions [165]. Hybrid-type fragmentation techniques such as electron-transfer/higher-energy collision dissociation (EThcD) can deliver informative product ions from both dissociation techniques [164]. However, EThcD takes more time to perform, limiting the cycle time and the overall number of product ion spectra that can be generated within an LC–MS/MS experiment and might not always be necessary to address the research question.

## Stage 4: data analyses and bioinformatics

Adequate software tools are the final key to successful glycoproteomics experiments. Different from many other types of PTMs, which can be considered as a single mass value that is either present or absent on the polypeptide chain, glycan modifications can range from a single monosaccharide residue to complex oligo- and polysaccharides, posing unique bioinformatics challenges. Like proteomics, the glycoproteome search space needs to be firstly appropriately defined. The current software for glycoproteomics differs in how the glycan search space is defined and incorporated into the process of glycopeptide identification [170]. Some tools allow not only the identification but also the relative or absolute quantitation of glycopeptides, and support annotation of sites of glycosylation [170]. The reliability of the software-based data analysis, however, is strongly influenced by the type and quality of the input data [166], the type of fragmentation method, and factors relating to the search engine and output filtering [171].

A suite of computational tools has been developed over the past decade, which have strongly contributed to the maturation and application of glycoproteomics (Table 2). While these informatics solutions have made impressive progress as summarised in recent reviews [39,103,170,172–174], some challenges remain. A recent inter-laboratory study conducted by the HUPO human glycoproteome initiative (HGI) to evaluate the performance of current software solutions and to identify high-performance search strategies for glycoproteomics data analysis, identified several high-performance software solutions, and at the same time demonstrated the significant informatics challenges that remain for glycopeptide data analyses — an important step forward to improve glycoproteomics software performance [171]. We expect some exciting new developments in this space in the coming years, supported by this and other community efforts [171,175,176] and the active integration of multi-dimensional data from different -omics technologies [177] (Figure 2). In the authors’ experience, the ability to incorporate glycomics data into the glycoproteomics workflow, coined as “glycomics-assisted glycoproteomics”, is an example of a particular useful integration of multiple-omics data sets. These allow an informed definition of the glycan search space whilst providing detailed information on the attached glycan structures [79,126,129,141,178–180]. Notably, careful manual review of the data output is still needed to obtain reliable and reproducible results in large-scale glycopeptide data analysis.

**Table 2.** Examples for commonly used and recently developed software tools for glycopeptide data analysis (in alphabetical order)

Software and access	Availability and integration (current version*)	Glycopeptide search strategy and key features	Compatible file types
*Byonic [* 205 *]* https://proteinmetrics.com/byos/	Commercial Regularly maintained and updated Can run as a stand-alone MS/MS-based search engine or as a node in Proteome Discoverer v1.4 or higher (Thermo) or Byos (PMI); [v4.0] Released in 2013	*De novo* intact *N*- and *O*-glycopeptide identification based on MS/MS data Handles all common types of fragmentation data but shows better performance for high-resolution HCD- and EThcD-MS/MS data Allows highly customisable searches Users can select variable modifications and glycan/protein search space Outcomes and search times benefit from prior knowledge of the sample investigated	Mgf Thermo raw mzML mzXML
*GlycoBinder [* 206 *]* https://github.com/IvanSilbern/GlycoBinder	Freeware Developed in R v3.5.00 Uses several (free) external tools, such as pGlyco v2.0 Released Oct. 2020; [v1.0.0]	Integrated in SugarQuant MS pipeline Allows quantification of TMT-labelled glycopeptides using MS3 data Combination of MS2 and MS3 scan data for confident glycopeptide identification from complex samples (reduced co-isolation of other precursor glycopeptide ions)	Thermo raw
*GlycoPAT [* 207 *]* https://virtualglycome.org/glycopat	Freeware Last update 2021 MATLAB v8.2 based Released in 2017; [v2.0]	Considers peptide and glycan fragmentation to calculate false discovery rate (FDR) scoring Can handle CID-MS/MS and other types of fragment data Modular tool, allowing more control over all phases of analysis, or integration of other tools at any point	mzML dta
*GlyXtool^MS^[* 208 *]* https://github.com/glyXera/glyXtoolMS	Freeware Developed in python v2.7 Released in 2018; [v2.0]	Modular tool, allowing control over all phases of analysis Allows filtering of spectra based on oxonium ions Suitable for analyses of moderately complex samples Open code allows further improvement of the pipeline, e.g. calculating FDR or including glycopeptide spectral matching, by modifying current tools or implementing new ones	mzML
*GPQuest [* 209 *]* https://www.biomarkercenter.org/gpquest	Freeware Last update 2019 MATLAB based Released in 2015; [v2.1]	*N*-glycopeptide analyses Needs to use library of deglycopeptides to perform identification HCD glycopeptide spectra containing oxonium ions are isolated before analyses, and compared with the previously generated library of glycosite-containing deglycopeptides Glycan assignment made by mass difference	mzML
*IQ-GPA (GlycoProteome Analyzer) [* 210 *]* https://www.igpa.kr	Freeware Web-based interface or desktop standalone (Windows only) Released in 2016; [v2.0]	*N*-glycopeptide analyses Can handle HCD-/CID-/EThcD-MS/MS data FDR calculation similar to GlycoPAT	Thermo or Bruker raw
*MetaMorpheus [* 211 *]* https://github.com/smith-chem-wisc/MetaMorpheus	Freeware Released Oct. 2020; [v.0.0.317]	*O*-Pair search methodology allows to improve site-specific identification, using paired HCD- and EThcD-MS/MS spectra from LC–MS/MS data Uses an ion-indexed search algorithm to improve speed and sensitivity of *O*-glycopeptide analyses, similar to MS-Fragger-Glyco Accepts user *O*-glycan databases	Mgf Thermo raw mzML
*MS-Fragger-Glyco [* 212 *]* https://msfragger.nesvilab.org	Freeware (Academic) or Commercial MSFragger can be used as a standalone software or integrated in Proteome Discoverer v2.2, 2.3 and 2.4 Released Nov. 2020; [v3.2]	Glycopeptide identification through open search or mass-offset Uses ion-indexed search algorithms adapted specifically to the properties of glycans to improve processing time as well as glycopeptide annotation	mgf (limited support) mzXML mzML
*pGlyco [* 213 *]* http://pfind.ict.ac.cn/software/pGlyco1505/	Freeware Last update 2020 Released in 2016; [v2.2.2]	Identification and annotation of intact *N*-glycopeptides which considers the glycan, peptide, and glycopeptide quality Limited to mammalian *N*-glycan search using sHCD pGlyco3 (in development) also allows to use ETD-, EThcD- and ETciD-MS/MS spectra, and introduces a new algorithm (pGlycoSite) to locate glycosylation sites (https://github.com/pFindStudio/pGlyco3/releases)	mgf
*Protein Prospector [* 214 *]* https://prospector.ucsf.edu/prospector/mshome.htm	Freeware Last update 2020 Web-based; [v6.2.2]	Identification of PTMs and modification sites Particularly suited for identifying *O*-glycopeptide sites using ET(hc)D-MS/MS data Less user-friendly interface	Mgf mzML

Important features of each software are briefly presented. Several software can be used to convert data, as in the case of generating mzML files using MSConvert included in ProteoWizard [215].

* As of June 2021.

## Stage 5: good reporting practice of glycoproteomics data

Data sharing and detailed reporting of MS-based glycoproteomics have become common practice [181,182], providing an opportunity for independent community review and data re-interrogation, but also a valuable resource for other researchers and software developers. However, with the increasing complexity and the enormous amount of data collected within a single experiment, the lack of a detailed and accurate reporting of experimental conditions limits the use by other scientists.

This was recognised several years ago for proteomics and led to the development of essential reporting guidelines (MIAPE) [183]. These guidelines have set an important standard that needs to be followed when submitting LC–MS/MS proteomics data to any of the data repositories under the ProteomeXchange consortium [182]. While many of the important experimental aspects for a glycoproteomics experiment are covered by the MIAPE guidelines, several key aspects of the glycan moiety of glycopeptides require particular attention in the reporting process. The Minimum Information Required for A Glycomics Experiment (MIRAGE) consortium has developed many guidelines (https://www.beilstein-institut.de/en/projects/mirage/) focussed on requirements for glycomics experiments that also contain aspects relevant to MS-based glycoproteomics experiments [184–187]. These guidelines are continuously being updated based on community feedback to facilitate the comprehensive reporting of glycomics experimental conditions, with dedicated glycoproteomics guidelines currently being drafted.

To facilitate sharing of glycomics and glycoproteomics data, the GlycoPOST (https://glycopost.glycosmos.org/) platform has been established as a data repository that supports the storage of MS, LC and LC–MS glycomics raw and analysed data, in addition to glycoproteomics data [188].

Several international efforts have been actively connecting glycomics and glycoproteomics data with other relevant glycobiological information to make current glycoproteomics knowledge more accessible. Initiatives such as Glyconnect (https://glyconnect.expasy.org/) [189], Glycomics@Expasy (www.expasy.org), GlyCosmos (https://glycosmos.org/) [190] and GlyGen (https://www.glygen.org/) [191] (see also http://www.glyspace.org/ [192]) have started to systematically curate glycoproteomics and glycomics data while linking information across each platform. These communal efforts will significantly facilitate the integration of glycoproteomics data into other -omics research resources.

## Conclusion

This review provides a concise overview of the methods now available for glycoproteomics analyses, with the intention to inform researchers that are new to the field, as well as experienced proteomics scientists that are considering jumping into the exciting wild waters of glycoproteomics. Glycoproteomics technologies have experienced a tremendous evolution over the past two decades, starting from the profiling of single glycoproteins, to now allowing large-scale system-wide analyses of complex samples as an integral part of multi-omics studies. Transcriptomics of glycosylation pathway relevant enzymes informs on how these pathways could be affected under studied conditions, metabolomics delivers important information on glycosylation precursors such as nucleotide sugar substrates that, in concert with (glyco)lipidomics and proteoglycomics, proteomics, glycoproteomics and genomics, can deliver a detailed picture of the highly interconnected cellular glycosylation pathways and how these are affected in diseases (Figure 2). These exciting developments will undoubtedly lead to an increased understanding of the function of glycoproteins in health and disease. It is also clear that these technologies are opening a new era in glycoscience that will, in combination with the other *-omics* techniques, deliver previously overlooked functional insights into the ubiquitous modification of proteins by glycans. Glycoproteomics analysis is and will increasingly become an indispensable part of understanding the molecular basis of life.

## Perspectives

- Glycoproteomics is becoming a powerful tool in glycobiology that enables the system-wide mapping of protein-specific glycosylation features.
- Understanding of protein-specific glycosylation and how it is impacted in diseases provides novel opportunities for precision diagnostics and therapies.
- Integration of glycoproteomics (and glycomics) into multi-omics studies is important to capture the glyco-language of cells and organisms.

## Open Access Statement

Open access for this article was enabled by the participation of Griffith University in an all-inclusive Read & Publish pilot with Portland Press and the Biochemical Society under a transformative agreement with CAUL.

## Abbreviations

CDGs: congenital disorders of glycosylation
CID: collision-induced dissociation
EThcD: electron-transfer/higher-energy collision dissociation
FASP: filter-aided sample preparation
HCD: higher-energy collisional dissociation
LAC: lectin affinity chromatography
PTMs: protein post-translational modifications
RP: reversed phase
TMT: tandem mass tags
